# *Hypericum perforatum*-induced hepatotoxicity with possible association with copaiba (*Copaifera langsdorffii Desf*):case report

**DOI:** 10.1590/S1679-45082014RC2953

**Published:** 2014

**Authors:** Marjorie Costa Agollo, Sender Jankiel Miszputen, Jayme Diament

**Affiliations:** 1 Escola Paulista de Medicina, Universidade Federal de São Paulo, São Paulo, SP, Brazil.; 2 Hospital Israelita Albert Einstein, São Paulo, SP, Brazil.

**Keywords:** Drug-induced liver injury, *Hypericum*/adverse effects, Phytotherapeutic drugs/adverse effects, Case reports

## Abstract

We report a case of liver damage in an elderly patient after the use of herbal products of *Hypericum perforatum* and *copaiba* (*Copaifera langsdorffii Desf*). Hepatotoxicity related to *Hypericum perforatum* is anecdotally known, but for copaiba, widely used as anti-inflammatory, there is just experimental data in the national literature. This report aimed to draw attention to the possible toxic effects of this association as well as to the clinical recovery of the patient after discontinuing their use. There is a tendency to suspect of the action of drugs to justify a non-viral acute liver injury, because of the large number of drugs responsible for hepatotoxicity. There are experiments and clinical reports in the literature describing some herbal products, including *Hypericum perforatum*, as the causative agents of this aggression, and are considered innocuous and used with no restrictions. We must remember that adverse reactions also occur with these substances; hence, they should be investigated when collecting the patient´s history, for leading to severe liver failure.

## INTRODUCTION

The liver metabolizes many medications and is therefore subject to drug-induced toxicity. Hepatotoxicity occurs due to direct action in case of tuberculostatic agents,^(1)^ statins and paracetamol,^(2)^ or it may be idiosyncratic and unexpected; it is an important clinical problem that may trigger liver failure, with fatal consequences, or patients may require urgent liver transplant.

The incidence of hepatotoxicity is considered underestimated since the use of some drugs is often omitted. It manifests as acute or chronic liver failure and represents the single cause in up to 30% of acute hepatitis, or it appears as cholestasis, steatosis or fibrosis. Over 900 exogenous substances with hepatotoxic potential have been identified and there are also reports of herbaceous plants that can cause harm.^(3)-6)^

Men use a large amount of foods, medications and dietary supplements derived from plants. Kennedy and Wightman^(7)^ revised the mechanisms of action of different phytotherapeutic agents: alkaloids (caffeine and nicotine), terpenes (ginkgo, ginseng, valerian and *Melissa officinalis*) and phenolic compounds (curcumin, isoflavone and *Hypericum perforatum*).

The acute clinical picture varies from no symptoms and abnormal laboratory findings found by chance to nausea, vomiting, abdominal pain, jaundice and acute hepatic failure, and it can overlap with preexisting liver diseases. The lesions are classified as hepatocellular, cholestatic, mixed or vascular. It is essential to ask about the use of medications to make diagnosis since there is no gold standard test, specific serum marker or histological profile to identify the cause of hepatic aggression.

Treatment consists of discontinuation of the suspected product, general precautions, relative rest and bland diet, in addition to monitoring liver function due to risk of progressing to hepatic failure.^(8)^

## CASE REPORT

A 79-year-old female patient sought care for presenting jaundice for one month. Past history of hypothyroidism. She was on *Hypericum* *perforatum*, copaiba, levothyroxine, omega 3, glucosamine and chondroitin. Upon physical examination, she was afebrile, normal blood pressure, with jaundice and tachycardia (100 bpm). Flacid abdomen, not painful upon palpation, no enlarged organs or signs of chronic liver disease. The laboratory tests revealed abnormal enzyme levels and hyperbilirubinemia, and other parameters were unaltered. Serology for viral hepatites and autoantibodies were negative. Magnetic resonance cholangiography showed no abnormalities.

Treatment prescribed was rest and withdrawing all medications, except hormone replacement for thyroid.

Among the laboratory tests that were initially increased, ALT-GPT dropped from 1,667U/L to 36U/L, and AST-GOT from 1,599U/L to 30U/L, after approximately 7 weeks. Similar recovery was observed in total bilirubin levels, that reduced from 9.0mg/dL to 1.24mg/dL, due to drop in the direct bilirubin fractions from 8.3mg/dL to 0.73mg/dL.

## DISCUSSION

*Hypericum perforatum*, or St John´s wort, is broadly distributed in many countries and indicated for treatment of mild to moderate depression,^(9)^ anxiety, insomnia and nevralgia, and its antiviral, antibacterial and photosensitizing activities are well known. There is no consensus about its antidepressant action,^(10)^ but in some countries, including Germany, it represents roughly 25% of all prescriptions of this drug class, and it is available in compounding pharmacies, and phytotherapeutic and natural product shops.

Acting on cytochrome P450, it might reduce the serum levels of some drugs or increase their clearance.^(3, 5)^ The interaction with other drugs favors its adverse effects,^(11, 12)^ including hepatotoxicity, as already mentioned.^(13)^

In the case presented, it was crucial to investigate other causes of acute hepatic disease, such as serology for hepatitis A, B and C viruses; autoimmunity despite her age, evaluated by the initial measurement of serum globulins; as well as imaging methods, particularly magnetic resonance cholangiography to exclude abnormalities in the intrahepatic and extrahepatic biliary ducts.

The laboratory follow-up of the patient showed progressive drop in enzyme and bilirubin levels, after discontinuing the drugs suspected of being hepatotoxic, and the tests returned to normal levels with no intercurrent events.

The second herbaceous product – copaiba – is an oil-resin extracted from the trunk of *Copaifera ssp*., and is often used in the Brazilian Northern and Northeastern regions as an anti-inflammatory agent. Its hepatotoxic effect is subject to discussion^(14)^ and its participation, together with another phytotherapeutic drug, is not excluded in triggering the described medication-induced acute hepatitis.

## References

[1] Malla I, Fauda M, Casanueva E, Fernández MI, Amante M, Cheang Y (2012). Fulminant hepatic failure due to tuberculostatic drugs: case report. Arch Argent Pediatr.

[2] O’Neil CK, Hanlon JT, Marcum ZA (2012). Adverse effects of analgesics commonly used by older adults with osteoarthritis: focus on non-opioid and opioid analgesics. Am J Geriatr Pharmacother.

[3] Amorim MF, Diniz MF, Araújo MS, Pita JC, Dantas JG, Ramalho JA (2007). The controvertible role of kava (Piper methysticum G. Foster) an anxiolytic herb, on toxic hepatitis. Rev Bras Farmacogn.

[4] Carvalho RJ, Khoury-Siyoufi ST, Kemp VL, Miszputen SJ (2007). Lesões hepáticas induzidas por drogas. Guias de medicina ambulatorial e hospitalar da UNIFESP-Escola Paulista de Medicina.

[5] Cordeiro CH, Chung MC, Sacramento LV (2005). Interações medicamentosas de fitoterápicos e fármacos: Hypericum perforatum e Piper methysticum. Rev Bras Farmacogn.

[6] Yellapu RK, Mittal V, Grewal P, Fiel M, Schiano T (2011). Acute liver failure caused by ‘fat burners’ and dietary supplements: a case report and literature review. Can J Gastroenterol.

[7] Kennedy DO, Wightman EL (2011). Herbal extracts and phytochemicals: plant secondary metabolites and the enhancement of human brain function. Adv Nutr.

[8] Chang CY, Schiano TD (2007). Review article: drug hepatotoxicity. Aliment Pharmacol Ther.

[9] Nahas R, Sheikh O (2011). Complementary and alternative medicine for the treatment of major depressive disorder. Can Fam Physician.

[10] Rapaport MH, Nierenberg AA, Howland R, Dording C, Schettler PJ, Mischoulon D (2011). The treatment of minor depression with St. John’s Wort or citalopram: failure to show benefit over placebo. J Psychiatr Res.

[11] Izzo A, Ernst E (2009). Interactions between herbal medicines and prescribed drugs: an updated systematic review. Drugs.

[12] Zhou SF, Lai X (2008). An update on clinical drug interactions with the herbal antidepressant St. John’s wort. Can Drug Metab.

[13] Domínguez Jiménez JL, Pleguezuelo Navarro M, Guiote Malpartida S, Fraga Rivas E, Montero Alvarez JL, Poyato González A (2007). Hepatotoxicity associated with Hypericum (St.John’s wort). Gastroenterol Hepatol.

[14] Noguchi A, Reis JM, Dias CS, Epaminondas WA, Azevedo PS, Brito MV (2002). Níveis séricos de aminotransferases, bilirrubinas e gama-glutamil transpeptidase após a administração de óleo de copaíba em ratos. Acta Cir Bras.

