# Supplementary material for: Hypericum perforatum-induced hepatotoxicity with possible association with copaiba (Copaifera langsdorffii Desf):case report
Source: Einstein (Sao Paulo). 2014 Jul-Sep;12(3):355–7. doi: 10.1590/S1679-45082014RC2953 (PMC4872950; doi:10.1590/S1679-45082014RC2953)
Supplement: Translation

# Translation: Hepatotoxicidade induzida por *Hypericum perforatum* com possível associação a copaíba (*Copaifera langsdorffii Desf*): relato de caso

**Authors:** Marjorie Costa Agollo, Sender Jankiel Miszputen, Jayme Diament

**Affiliations:** 1 Escola Paulista de Medicina, Universidade Federal de São Paulo, São Paulo, SP, Brasil.; 2 Hospital Israelita Albert Einstein, São Paulo, SP, Brasil

**Keywords:** Doença hepática induzida por droga, *Hypericum*/efeitos adversos, Medicamentos fitoterápicos/efeitos adversos, Relatos de casos

## Abstract

Relatamos um caso de dano hepático em paciente idosa após uso de produtos naturais à base de *Hypericum perforatum* e copaíba (*Copaifera langsdorffii Desf*). A hepatotoxicidade do *Hypericum perforatum* é conhecida por relatos, mas a respeito da copaíba, largamente usada como anti-inflamatório, há apenas dados experimentais na literatura nacional. Essa apresentação visou chamar a atenção para possível efeito tóxico dessa associação, bem como para a recuperação clínica da paciente após interrupção de seu uso. Há uma tendência de suspeitar sobre a ação de medicamentos para justificar a lesão hepática aguda não viral, em razão do grande número de fármacos responsáveis por hepatotoxicidade. A literatura tem publicado experimentos e relatos clínicos em que produtos fitoterápicos, inclusive *Hypericum perforatum*, são o agente causador dessa agressão, considerados inócuos e utilizados livremente. É preciso lembrar que reações adversas ocorrem também com essas substâncias, merecendo sua investigação na obtenção da anamnese, pela possibilidade de promoverem quadros graves de falência hepática.

## INTRODUÇÃO

O fígado metaboliza grande número de medicamentos, estando sujeito, desse modo, à toxicidade induzida por eles. Sua hepatotoxicidade ocorre por ação direta, no caso de tuberculostáticos,^(1)^ estatinas, paracetamol,^(2)^ ou é idiossincrásica e inesperada, constituindo um problema clínico importante, que pode promover falência hepática, com consequências fatais, ou requerer transplante de urgência.

Acredita-se que a incidência seja subestimada, pois, usualmente, seu uso é omitido. Manifesta-se de forma aguda ou crônica, sendo a única causa em até 30% das hepatites agudas, ou como colestase, esteatose ou fibrose. Estão identificadas mais de 900 substâncias exógenas com potencial hepatotóxico, bem como relatos com plantas herbáceas.^(3)-6)^

O homem utiliza extensa quantidade de alimentos, medicamentos e suplementos dietéticos derivados de plantas. Kennedy e Wightman^(7)^ revisaram os mecanismos de ação de diferentes fitoterápicos: alcaloides (cafeína e nicotina), terpenos (ginkgo, ginseng, valeriana e Melissa officinalis) e fenólicos (curcumin, isoflavona e Hypericum perforatum).

O quadro clínico agudo varia desde alterações laboratoriais reconhecidas ao acaso e sem nenhum sintoma, até náuseas, vômitos, dor abdominal, icterícia e insuficiência hepática aguda, podendo se sobrepor à hepatopatias preexistentes. As lesões são classificadas em hepatocelular, colestática, mista ou vascular. Inquerir sobre uso de drogas é fundamental para o diagnóstico, pois não há exame padrão-ouro, marcador sérico específico e nem perfil histológico para identificação daquela responsável pela agressão hepática.

O tratamento consiste em suspender o produto suspeito, tomar medidas gerais, fazer repouso relativo e dieta branda, além de monitorar a função hepática, pelo risco de evolução para insuficiência hepatocelular.^(8)^

## RELATO DE CASO

Mulher, 79 anos, atendida por apresentar icterícia há 1 mês. História prévia de hipotireoidismo. Em uso de Hypericum perforatum, copaíba, levotiroxina, omega 3, glucosamina e condroitina. Ao exame físico, apresentava-se afebril, ictérica, normotensa e taquicárdica (100bpm). Abdome flácido, indolor, sem visceromegalias ou sinais de hepatopatia crônica. Exames laboratoriais evidenciaram alterações enzimáticas e hiperbilirrubinemia, com outros parâmetros preservados. As sorologias para hepatites virais e autoanticorpos foram negativas. Colangiorressonância sem alterações.

O tratamento instituído foi repouso e suspensão de todas as medicações, exceto a reposição hormonal para a tireoidopatia

Dentre os parâmetros laboratoriais inicialmente elevados, a ALT-TGP de 1.667U/L, após cerca de 7 semanas, caiu para 36U/L, e a AST-TGO de 1.599U/L para 30U/L. Recuperação semelhante foi observada nos níveis de bilirrubina total, que passaram de 9,0mg/dL para 1,24mg/dL, em razão da queda das taxas da fração direta, de 8,3mg/dL para 0,73mg/dL.

## DISCUSSÃO

O Hypericum perforatum, ou Erva de São João, é largamente distribuído em vários países, com indicação para tratamento de depressão leve a moderada,^(9)^ ansiedade, insônia e dores nevrálgicas, tendo reconhecida atividade antiviral, antibacteriana e fotossensibilizadora. Sua ação antidepressiva não é consensual,^(10)^ mas, em alguns países, como na Alemanha, representa cerca de 25% do total de prescrições dessa classe de medicamento, estando acessível em farmácias de manipulação, de fitoterápicos ou de produtos naturais.

Agindo sobre o citocromo P450, pode reduzir os níveis séricos de alguns medicamentos ou aumentar seu clareamento.^(3, 5)^ A interação com outros fármacos favorece seus efeitos adversos,^(11, 12)^ sendo a hepatotoxicidade já descrita.^(13)^

No caso apresentado, foi imperativo pesquisar outras causas de doença hepática aguda: sorologias para vírus das hepatites A, B e C; autoimunidade, apesar da idade, avaliada pela dosagem inicial das globulinas séricas; e métodos de imagem, particularmente colangiorressonância, para excluir anormalidades nas vias biliares intra e extra-hepáticas.

A evolução laboratorial da paciente mostrou diminuição progressiva das enzimas e da bilirrubina, após a suspensão das drogas suspeitas da hepatotoxcidade, com sua normalização sem outras intercorrências.

O segundo produto herbáceo, a copaíba, um óleo-resina extraído do tronco da Copaifera ssp., muito utilizado como anti-inflamatório nos Estados das Regiões Norte/Nordeste do Brasil, tem seu efeito hepatotóxico discutível,^(14)^ não se excluindo a possibilidade de sua participação, em conjunto com o outro fitoterápico, no desencadeamento da hepatite aguda medicamentosa descrita.

## CONCLUSÃO

Esta apresentação procurou reforçar a importância de uma história clínica detalhada, com especial atenção às medicações utilizadas pelos doentes. A inocuidade aparente de produtos naturais permite seu livre consumo, sem considerá-los de risco para o desenvolvimento de doenças. O mesmo motivo faz o doente omitir essa informação em sua história clínica. Cabe ao médico questionar sobre todas as drogas em uso atual ou recente, investigar sobre suas características de toxicidade, especialmente quando da interação com outros medicamentos, pois terá um maior conjunto de dados para a hipótese diagnóstica e sua diferenciação. O que é válido para a exploração de qualquer doença é particularmente importante nos casos das hepatopatias, já que essa víscera mostra especial sensibilidade na resposta à ação química de determinados fármacos. Particularmente no presente caso, deu-se ênfase ao uso da copaíba associado ao Hypericum.
